# Highly sensitive and specific responses of shrimp gill cells to high pH stress based on single cell RNA-seq analysis

**DOI:** 10.3389/fcell.2022.1031828

**Published:** 2022-11-08

**Authors:** Qianqian Ge, Jiajia Wang, Jitao Li, Jian Li

**Affiliations:** ^1^ Laboratory for Marine Fisheries Science and Food Production Processes, Pilot National Laboratory for Marine Science and Technology (Qingdao), Qingdao, China; ^2^ Key Laboratory of Sustainable Development of Marine Fisheries, Ministry of Agriculture and Rural Affairs, Yellow Sea Fisheries Research Institute, Chinese Academy of Fishery Sciences, Qingdao, China

**Keywords:** gill, shrimp, scRNA-seq, high pH stress, cell heterogeneity

## Abstract

High pH is one of the main stressors affecting the shrimp survival, growth, and physiology in aquaculture ponds, but the cellular and molecular mechanism responsible for high pH stress has not been elucidated in shrimp. In this study, the shrimp acid-base disturbance and gill cell alterations were significantly observed and then single cell RNA-sequencing (scRNA-seq) was performed to study the sensitive and specific responses of gill cells to high pH stress. Three main gill cell types, including pillar cells, hemocytes and septal cells were identified. By comparative scRNA-seq analysis between control and pH group, the pillar cell was regarded as the target cell type in response to high pH stress with the down-regulation of ammonia excretion and H^+^ transport related genes and up-regulation of immune related genes. Notedly, high pH resulted in the emergence of a new immune cell subcluster in pillar cells, with immune activation and stress defense states. Pseudotime analysis also showed that the pillar cells could transform into the functionally inhibited ion cell subclusters and functionally activated immune cell subclusters after high pH stress. Further, the regulatory network of pillar cell population was predicted by WGCNA and two transcription factors were identified. In conclusion, these results provide key insights into the shrimp gill cell-type-specific mechanisms underlying high pH stress response at a single-cell resolution.

## Introduction

The pH is an important environmental factor affecting the life of aquatic crustaceans (Han et al., 2018a). In general, the effects of low pH on shrimps have received considerable attentions, however, the high pH caused by contamination of water, red tide, elevated photosynthesis rates that occur during the afternoon or saline-alkaline water is also very common in the shrimp farming pond and aquaculture systems (Li et al., 2021). Especially, the pH of water in aquaculture farms can increase dramatically (potentially reaching values greater than pH 9.5) due to microalgal over-multiplication during the middle and late rearing periods, thereby causing damage to the reared organisms (Lin et al., 2013). In aquaculture, the optimal pH suited to most aquatic animals is 7.8–8.5, once the pH exceeds 9.0, stress will occur (Wang et al., 2002; Li et al., 2019). Alkaline water environment may damage the gill directly, causing it to lose ion transport ability and impair respiration (Zhou et al., 2008). The high pH in alkaline water often negatively affects nitrogen waste excretion for aquatic animals (Wilkie and Wood, 1996). Additionally, high pH could trigger many stress responses in physiological status, biochemical level, and metabolism pathways in shrimps, leading to high mortality, oxidative stress, immune suppression, and pathogen susceptibility (Li and Chen, 2008; Liu et al., 2015; Han et al., 2018b; Huang et al., 2018). Although efforts have been made to expound the detrimental effects of high pH on shrimp, the molecular response mechanism under high pH stress in shrimp, especially the cellular response mechanism needed to deeply decipher.

The shrimp gill acts as a direct interface between hemolymph and the ambient environment, which are generally multifunctional, performing respiration, osmoregulation, acid-base balance, and excretion of nitrogenous end products while also acting as a major toxicological interface for metals and pollutants (McNamara and Faria, 2012; Allen and Weihrauch, 2021). Given the importance of gill, many omics methods such as transcriptome, proteome and metabolome have been applied to study their responses under pH stress at the ensemble, cell population levels (Wei et al., 2015; Li et al., 2020; Zhu S et al., 2022). However, there is a lack of consensus on the cell classification of gills, which markedly hinders our understanding of the detailed ion transport functions. Although the cell types of shrimp gills such as pillar cells, nephrocytes, fibroblasts, nerve cells, and glandular cells have been investigated by histological and physiological techniques, there is no universally accepted structural and functional conceptualization (Taylor and Taylor, 1992). Therefore, in order to further understand shrimp cell diversity and function, it is necessary to identify the cell types and the molecular signatures of all dynamic states of cell types under steady state and stress conditions.

Single-cell RNA sequencing (scRNA-seq) is a powerful tool for studying the state and function of cells. Recently, scRNA-seq has been used to reveal cellular characteristics and functions in several aquatic invertebrates, dramatically changing the understanding of the cellular diversity of multiple aquatic invertebrates, such as *Penaeus monodon*, *Litopenaeus vannamei*, *Crassostrea hongkongensis*, and scallops (Sun et al., 2021; Li et al., 2022; Meng and Wang, 2022; Zhu W. L et al., 2022). However, scRNA-seq has not previously been used to analyze the gill cells of aquatic animals. The ridgetail white prawn, *Exopalaemon carinicauda*, has great adaptive capacity to extreme environments, especially for pH, carbonate alkalinity and salinity (Shi et al., 2022). In recent years, *E. carinicauda* has successfully cultivated in saline-alkaline waters in China, which make it attractive as an excellent model for studying the response mechanism under high pH stress in crustacean (Ge et al., 2019). Here, we performed histological analysis and scRNA sequencing of gill in *E. carinicauda* under high pH stress, to comprehensively clarify the highly sensitive and specific responses of shrimp gill cells to high pH stress. This study focused on developing an atlas of diverse gill cell populations in shrimp to provide a valuable resource for the gene expression profiles of the various cell types. It also provided valuable information for revealing the cellular and molecular response mechanism of shrimp in response to high pH stress.

## Materials and methods

### Animals and high pH stress experiment

Healthy *E. carinicauda* (1.30 g ± 0.09 g) were obtained from the Yellow Sea Fisheries Research Institute in Qingdao (Shandong, China) and acclimated in 200 L polyvinyl chloride polymer (PVC) tanks containing aerated and sand-filtrated seawater (salinity 31 ppt, pH 8.0 ± 0.1, temperature 23°C ± 1°C) for 2 weeks. 240 shrimps were randomly selected and equally divided into two groups (named control and pH group) with three replicates each. A replicate was one 10 L PVC tank containing 20 shrimps. In the pH group, about 15 ml–20 ml NaOH solution (1 M) was added into 10 L seawater by inches until pH reached to 9.4 (LD_50_ for 72 h of high pH stress), and the salinity was still 31 ppt. Because the pH value would change rapidly in a short time during the stress process, the pH was adjusted hourly to maintain the pH at 9.4 to ensure relatively stable pH stress during the 72 h stress experiment. In the control group, pH was kept at 8.0. Hemolymph from four shrimps in each tank was collected as a pool for analyzing hemolymph pH at 1 h, 6 h, 12 h, 24 h, 48 h, and 72 h, respectively. At 48 h, gills from 6 shrimps per group were sampled to test scRNA and gills from 3 shrimps per group were sampled to test gene expression.

### Hemolymph pH measurement

Hemolymph was sampled from shrimp cardiocoelom by 1 ml sterile syringe, and the hemolymph pH was tested by a pH Tester (HI98103, HANNA, Italy). The remaining hemolymph was centrifuged at 4,000 g for 20 min at 4°C (Eppendorf centrifuge 5,810 R) to obtain serum. The serum was analyzed for osmolality. Serum osmolality was measured in undiluted 70 μl aliquots using a freezing point osmometer (SMC 30C-1, Tianhe, China).

### Transmission electron microscopy

Gills from shrimp in control group and pH group were fixed in 2.5% glutaraldehyde in 0.1 M phosphate buffer saline (PBS), pH 7.4, at 4°C for 24 h and were post-fixed by 1% OsO_4_ for 2 h. After dehydration in ascending graded ethanol (30%-50%-70%-80%-95%-100%–100% ethanol concentrations orderly, 20 min at a time) and clearness by 100% acetone twice (15 min at a time), tissues were molded with epoxy resin, kept in an incubator at 60°C for 48 h, cooled down, sectioned (60 nm–80 nm) through ultra-microtome (Leica ultracut UCT) with a glass knife, and stained by 2% uranium acetate saturated alcohol solution avoid light staining for 8 min and 2.6% Lead citrate avoid CO_2_ (Arumugam et al., 2021).

### Preparation of gill single-cell suspensions

The fresh gills were immediately used for their single cell suspension. Briefly, after being washed 2–3 times with DPBS (1×PBS without calcium and magnesium, Na_2_HPO_4_ 8 mM, NaCl 136 mM, KH_2_PO_4_ 2 mM, pH 7.8) and cut into about 1 m^3^ small pieces, the gills were successively digested with collagenase (2 mg/ml) and disperse enzyme (0.2 mg/ml) at 37°C for 15 min. The digested solution was centrifuged to collect the cell sediment and the pre-cooled CMFSS (calcium free magnesium ion buffer, 25.5 g/L NaCl, 0.8 g/L Na_2_SO_4_, and 2.86 g/L HEPES) was used to separate gill cells in the cell sediment (Sun et al., 2021). Subsequently, the cell suspension was filtered through the 40 μm cell strainer to remove large particles, and then centrifugated at 500 g for 10 min. Cell viability and number were assessed by 0.4% trypan blue staining and cell counting using cell counting plate. High-quality cell suspension of gill cells was subjected to single cell encapsulation by 10X Genomics v3 kit (10x Genomics, United States).

### Single-cell RNA sequencing

Cell density were adjusted to approximately 1,000 cell/μl. Gel beads containing barcode information were bound to the mixture of cells and enzymes, and bound molecules were then wrapped in oil surfactant droplets using the microfluidic “double cross” system to create single-cell Gel Beads-In-Emulsions (GEMs). The GEMs flowed into the reservoir and were collected, the gel beads were dissolved to release the barcode sequences, and the cDNA fragments were constructed by reverse transcription and global amplification in a Mastercycler Pro thermocycler (Eppendorf, Hamburg, Germany). The cDNA sequences were used as templates for PCR amplification, and the PCR amplicons were mixed to construct a sequencing library. The cDNA libraries were sequenced on the Illumina novaseq 6000 platform. The BioProject number submitted to NCBI is PRJNA882579.

### Cell clustering

Due to the genome of *E. carinicauda* is still unknown, the full-length transcriptome data was prepared as a reference for mapping scRNA-Seq Data (NCBI accession number: PRJNA594425) (Shi et al., 2020). 10X Genomics Cell Ranger software (version 3.1.0) was used to align, filter and normalize the raw scRNA-seq data. Briefly, reads with low-quality barcodes and Unique Molecular Identifiers (UMIs) were filtered out and after the initial quality control screen by the Cell Ranger, the sequencing data were aligned to the reference transcriptome. Subsequently, the cell by gene matrices for each sample were individually imported to a popular R package Seurat for data normalization, cell clustering, marker gene screening, and gene expression analysis among different cell clusters. To start, cells with unusually high number of UMIs (≥ 8,000) or mitochondrial gene percent (≥ 10%) were filtered out and excluded cells with less than 500 or more than 4,000 genes detected. After removing of low-quality cells and normalizing data were normalized with the formula: A gene expression level = log [1+(UMI_A_/UMI_Total_) × 10,000]. Then, the normalized data were clustered using principal component analysis (PCA) and visualized with t-distributed Stochastic Neighbor Embedding (t-SNE). The cell subset was grouped by graph-based clustering based on the gene expression profile of each cell.

### Differentially Expressed Gene analysis

To explore molecular differences between control and pH group, we identified DEGs in two sets of comparisons using Seurat. The genes were considered significantly differentially expressed by the following criteria: |log_2_FC| ≥ 0.36, the proportion of cells expressing the target gene in a given cluster was ≥ 0.1, and ≥ 25% of all cells in that same cluster expressed the target gene. Then, the hurdle model in Model-based Analysis of Single-cell Transcriptomics (MAST) was used to identify the DEGs in each cluster. The Benjamini–Hochberg method in Seurat was used to correct *p*-values, and genes were considered DEGs when the corrected *p*-value was ≤ 0.05. Subsequently, functional enrichment analyses were performed for the DEGs. For GO enrichment analysis, DEGs were first mapped to the InterProScan database to obtain a list of genes with a certain GO function in each term, and significantly enriched GO terms were identified by a hypergeometric test with a threshold of *p*-value ≤ 0.05. For KEGG enrichment analysis, a Clusterprofiler package (v4.0.5) was applied to find pathways significantly enriched in DEGs compared to the background of the entire transcript set with a threshold of *p*-value ≤ 0.05.

### Pseudotime analysis

Monocle 2 was used to infer the pseudotemporal ordering of pillar cells. We assumed the raw UMI counts were distributed according to a negative binomial distribution with fixed variance expression family to model the raw UMI count data, as recommended by the authors of Monocle 2. The Monocle 2 function BEAM was used to identify pillar cells that were enriched along particular branches in the pseudotime tree. Branched heat maps were constructed using genes with *q*-values < 5 × 10^–5^ from the BEAM. The GO and KEGG enrichment of the DEGs in each branch were analyzed as described in Differentially Expressed Gene (DEG) Analysis.

### Weighted gene co-expression network analysis analysis of pillar cells

Weighted gene co-expression network analysis (WGCNA) package (v1.47) in R was used to construct co-expression networks by pillar cell subclusters. The modules were constructed by automatic network construction function blockwise-Modules with the settings: power 8, TOM Type unsigned, merge-CutHeight 0.7, min-ModuleSize 50, and remaining settings on default. Furthermore, to find out significant modules, module eigengenes were used to calculate the correlation coefficient with samples or sample traits. The intramodular connectivity (K.in) and module correlation degree (MM) of each gene were calculated by WGCNA, and genes with high connectivity tended to be hub genes which may have key functions. Cytoscape_3.3.0 was used to visualize the networks with transcription factors. For genes in selected module, GO and KEGG pathway enrichment were conducted to analyze the biological functions (Meng and Wang, 2022).

### Real-time quantitative PCR

The methods of RNA isolation, cDNA synthesis, and RT-qPCR assay and the primers were based on our previous studies (Ge et al., 2019).

## Results

### Hemolymph pH and gill ultrastructure alterations

In the control group, the hemolymph pH of *E. carinicauda* remained stable at 7.62–7.65. When the pH of external water environment changed, the hemolymph pH of *E. carinicauda* also changed. In the high pH stress group, the hemolymph pH fluctuated in the range of 7.83–8.14, showing a trend of first increase and then decrease. It reached the highest value of 8.14 at 48 h and then decreased slightly, but was still higher than the normal hemolymph pH at 72 h (Table 1).

**TABLE 1:** The hemolymph pH of *E. carinicauda* under pH stress.

Time pH		1 h	6 h	12 h	24 h	48 h	72 h
Control	pH_e_	7.95 ± 0.08^a^	7.92 ± 0.10^a^	8.02 ± 0.08^a^	8.04 ± 0.05^a^	7.93 ± 0.15^a^	8.01 ± 0.04^a^
	pH_h_	7.65 ± 0.04^a^	7.65 ± 0.03^a^	7.62 ± 0.03^a^	7.65 ± 0.02^a^	7.64 ± 0.05^a^	7.62 ± 0.01^a^
pH	pH_e_	9.20 ± 0.09^ab^	9.30 ± 0.07^abc^	9.45 ± 0.08^c^	9.40 ± 0.10^c^	9.20 ± 0.09^ab^	9.32 ± 0.07^bc^
	pH_h_	7.83 ± 0.02^a^	8.00 ± 0.05^bc^	8.10 ± 0.03^de^	8.05 ± 0.02^cd^	8.14 ± 0.04^e^	8.00 ± 0.02^bc^

Note: *pH*_*e*_ pH of water environment, *pH*_*h*_ pH of shrimp hemolymph. The meaning of the symbol a, b, c, d, and e indicated that values with different superscript letters in the same line are significantly different (*p* < 0.05).

**FIGURE 1:**
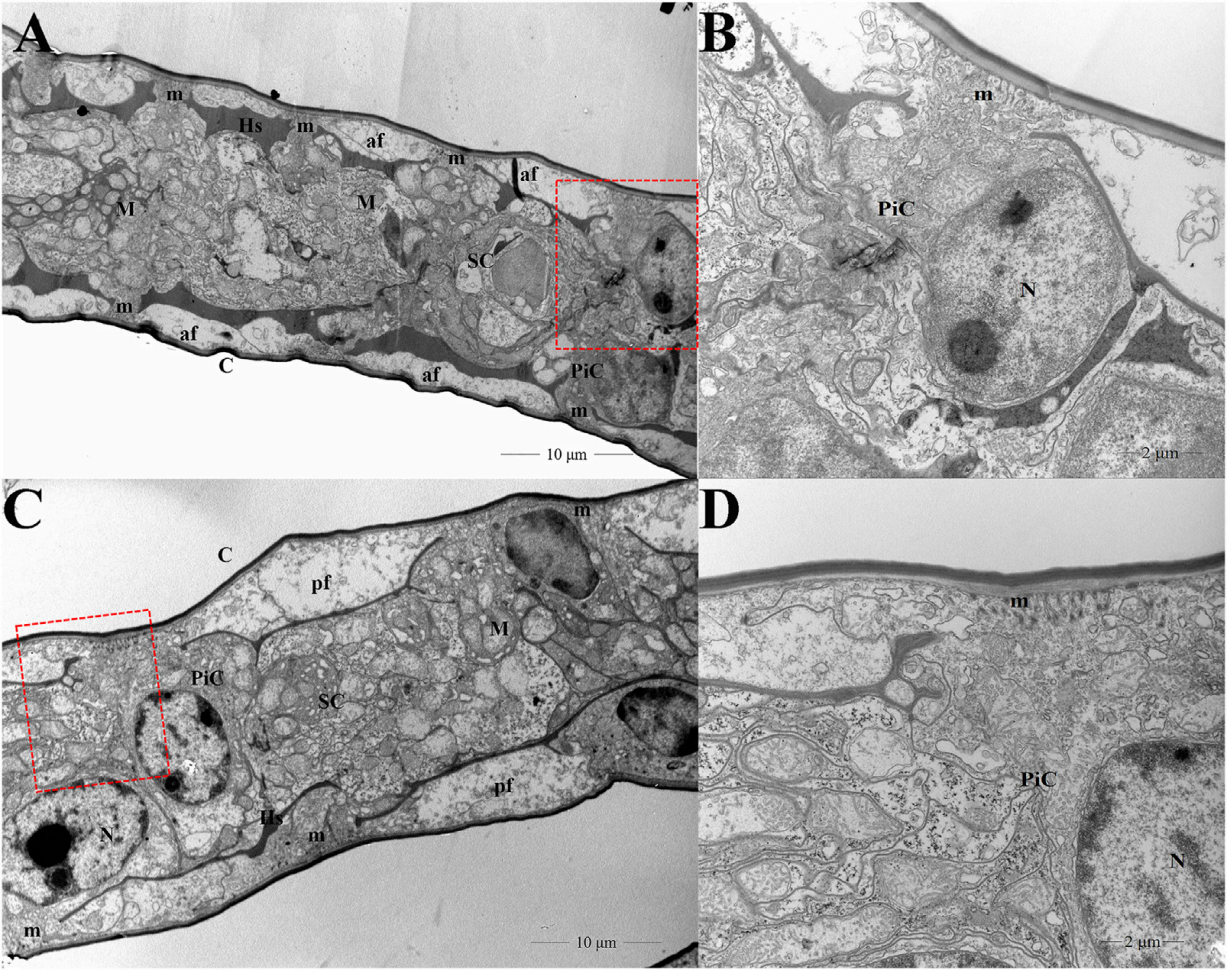
Gill ultrastructure of transverse sections in the control **(A,B)** and pH group **(C,D)**. Figures 1C,D were the enlargement of red frame in Figures 1A,B, respectively. *C* cuticle; *Hs* hemolymph space; *M* mitochondria; *N* nucleus; m microvillus; *PiC* pillar cells; *SC* septal cells; *af* apical flange.

Ultrastructural micrographs of *E. carinicauda* gills illustrated two main cell types: pillar cells and septal cells. A network of hemolymph space was located between the two cell types, where two rows of symmetrical space were visible on both sides of the septal cells. The pillar cells presented apical flanges that were lateral expansions extending under a thin cuticle and microvillus were present in the apical side of the flanges. The cytoplasmic membrane of septal cells was highly amplified by deep and numerous infoldings associated with abundant mitochondria. The pillar cells were distributed on either side of the septal cells. The hemolymph spaces were detected at the basal sides of the flanges of pillar cells, but hemocytes were not displayed in this figure (Figures 1A,B). After high pH stress, various ultrastructural modifications in the gills were induced. To be specific, the average thickness of the pillar cell flanges significantly increased compared to the control group. An apparent increase in the size and number of microvilluses in pillar cells was observed. In addition, the volume of hemolymph space was significantly decreased. We did not observe any modification in the septal cells between control and pH group (Figures 1C,D).

### Global transcriptome profile of gill cells

**FIGURE 2:**
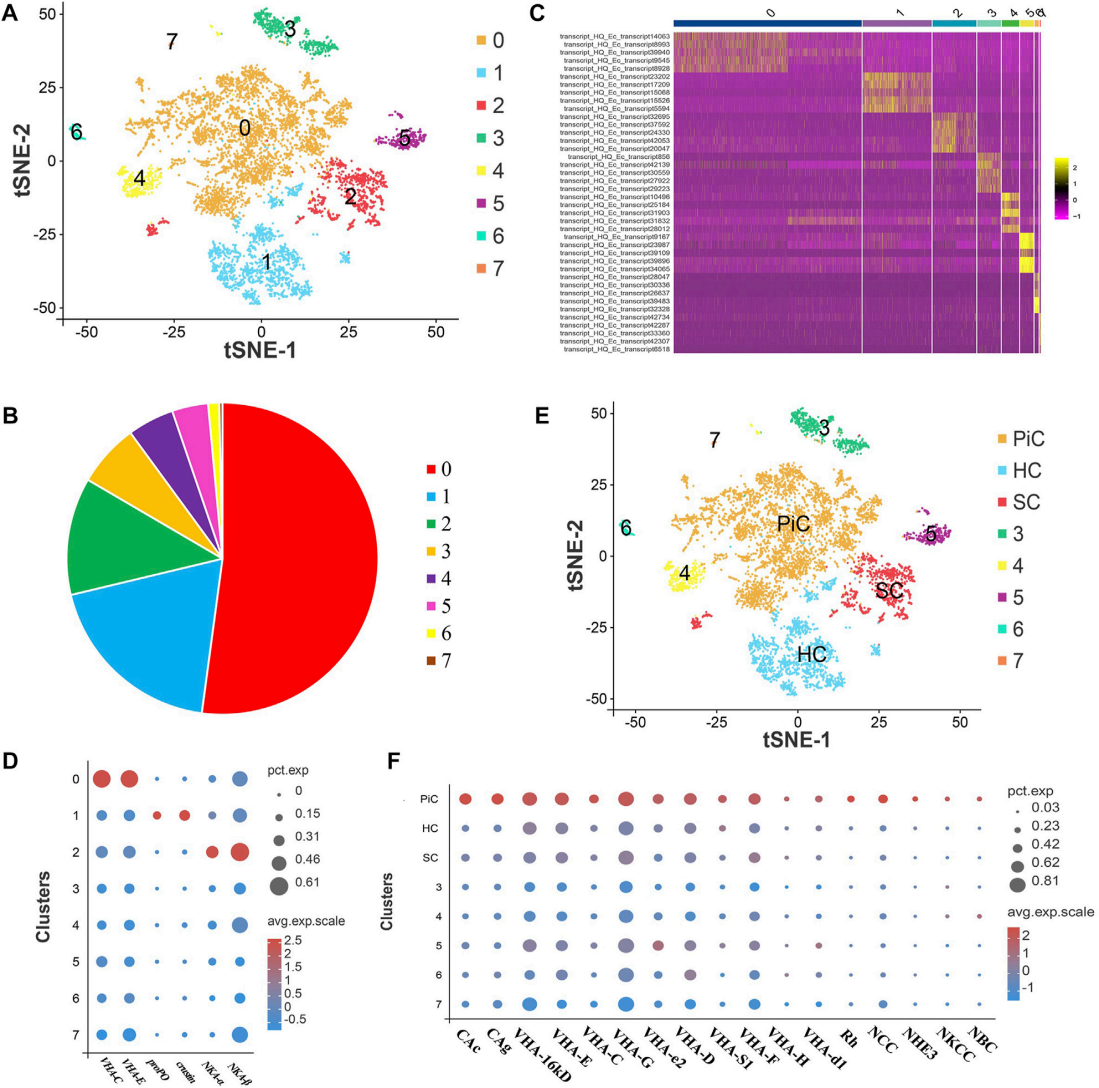
ScRNA-seq analysis of gill cells in *E. carinicauda*. **(A)** The t-SNE nonlinear clustering of gill cells in *E. carinicauda*. **(B)** The proportional distribution of each cell cluster in the total gill dataset. **(C)** Heatmap of the top 10 marker genes in gill cell clusters. **(D)** Dot plot representing the known marker genes of gill cells in clusters. **(E)** t-SNE visualization showing the cell names of gills. *PiC* pillar cells, *HC* hemocytes, *SC* septal cells. Cluster 0, 1 and 2 were identified as PiC, HC and SC, respectively. **(F)** Dot plot representing the distribution of ion transport related genes in clusters. *CAg* glycosyl-phosphatidylinositol-linked carbonic anhydrase, *CAc* cytoplasmic carbonic anhydrase, *VHA* V-type proton ATPase, *Rh* ammonium transporter Rhesus glycoprotein, *NCC* sodium-chloride cotransporter, *NHE3* Na^+^-H^+^ exchanger 3, *NKCC* Na^+^-K^+^-2Cl^-^ cotransporter, *NBC* sodium bicarbonate cotransporter.

After standard quality control dataset filters, eight cell populations (Cluster 0–7) were obtained that can be visualized using t-SNE (Figure 2A). A total of 8,115 cells (control: 4,731, pH: 3,384) from cell suspensions of whole gill cells were isolated and sequenced. These cell clusters consisted of 0.3%–52.1% cells, and cluster 0 accounted for half. Notedly, the cells in cluster 0, 1, and 2 accounted for more than 80% (Figure 2B). The expression levels and the percentage of cells expressing the top ten genes in each cluster are shown in a heat map, which indicated that each cluster had specific biomarkers (Figure 2C). GO enrichment analysis of the DEGs in cluster 0, 1, and 2 were performed. The GO terms in cluster 0 and Cluster 2 were both mainly related to ion transport, and the GO terms in cluster 1 were mainly related to response to stimulus (Supplementary Figure S1). According to gene expression markers from previous studies, the distinct types of three major cell clusters were defined, that is, cluster 0, 1, and 2 were identified as pillar cells, hemocytes, and septal cells, respectively (Figures 2D,E) (Maraschi et al., 2015; Koiwai et al., 2018; Lv et al., 2020). As displayed in dot plot, the ion transport related genes including carbonic anhydrases (CAc and CAg), V-type proton ATPases (VHAs), ammonium transporter Rhesus glycoprotein (Rh), sodium-chloride cotransporter (NCC), Na^+^-H^+^ exchanger 3 (NHE3), Na^+^-K^+^-2Cl^-^ cotransporter (NKCC) and sodium bicarbonate cotransporter (NBC) were found to be expressed dominantly in pillar cells (Figure 2F). The RT-qPCR results also showed high expression of some ion transport related genes in gills after high pH stress (Supplementary Figure S2).

### Comparative single-cell transcriptome analysis

**FIGURE 3:**
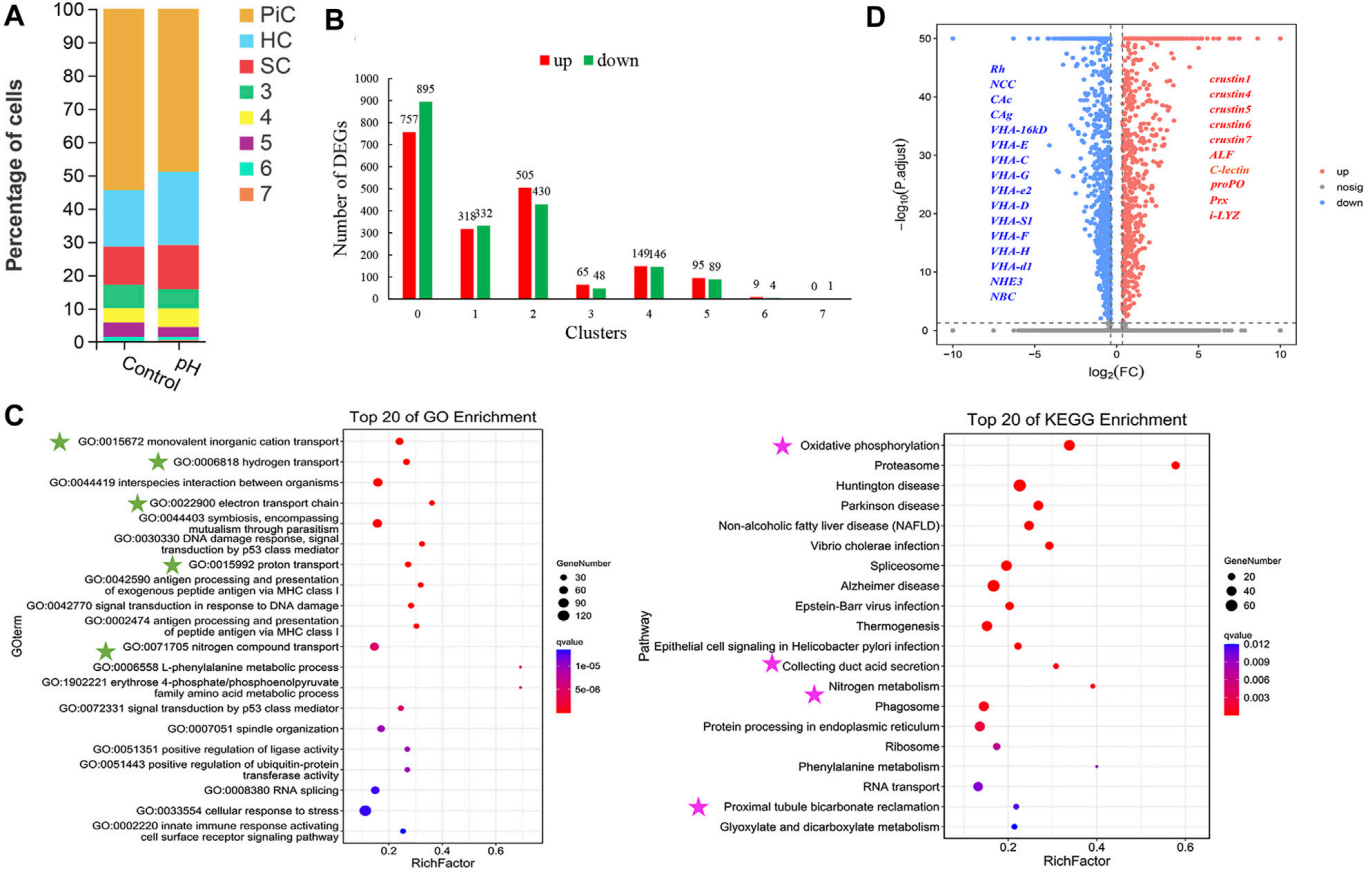
The changes of cell clusters under high pH stress and enrichment of DEGs. **(A)** The comparison of cell number ratios between control and pH groups. Cluster 0, 1 and 2 were identified as PiC, HC and SC, respectively. **(B)** Up- and down- regulated DEG numbers in each cell type of gills after high pH stress. **(C)** GO and KEGG enrichments of DEGs in pillar cells. The green stars indicated the GO terms related to ion transport, and the pink stars indicated the KEGG pathways related to ion transport. **(D)** Differential gene volcanic map display of pillar cells. *ALF* Anti-lipopolysaccharide factor, *proPO* prophenoloxidase, *Prx* peroxiredoxin, *i-LYZ* i-lysozyme.

The comparison of cell number ratios between control and pH groups showed that the pillar cell was decreased, and hemocyte and septal cell were increased after high pH stress, but the changes were not particularly significant (Figure 3A). A total of 3,843 DEGs were identified between control and pH group, and pillar cells contained the most DEGs than other cell types (Figure 3B). Further, GO and KEGG enrichment analyses of the marker genes with significant expression differences in control and pH group revealed that the DEGs in pillar cells were mainly related to ion transport. For GO terms in pillar cells, the DEGs were significantly enriched in “monovalent inorganic cation transport”, “hydrogen transport”, “electron transport chain”, “proton transport”, and “nitrogen compound transport”. For KEGG pathways in pillar cells, the DEGs were significantly enriched in “Oxidative phosphorylation” and “Collecting duct acid secretion” (Figure 3C). Notedly, the ion transport related genes in pillar cells such as VHAs, CAs, Rh, NCC, and NBC were down-regulated, and the immune related genes in pillar cells such as crustins, anti-lipopolysaccharide factor (ALF), C-type lectin, prophenoloxidase (proPO) and peroxiredoxin (Prx) were up-regulated after high pH stress (Figure 3D).

### Heterogeneous responses of pillar cells

**FIGURE 4:**
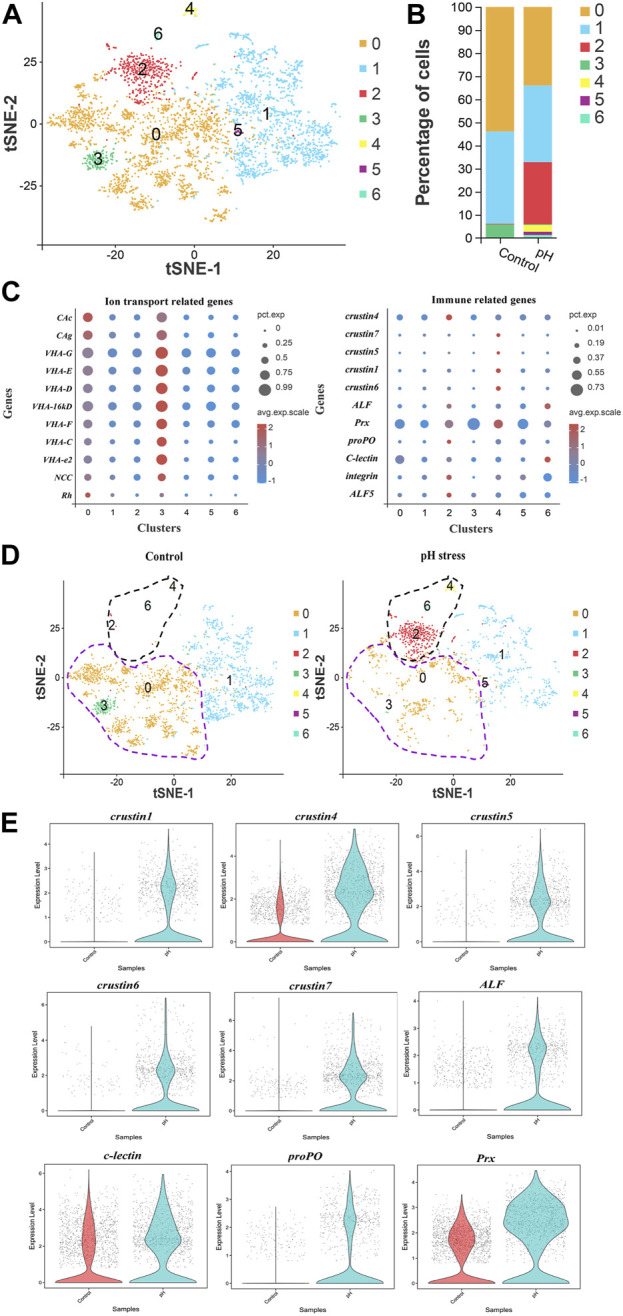
Heterogeneity of pillar cells between control and pH groups. **(A)** The t-SNE plot showing the seven subclusters identified in the pillar cells. **(B)** The percentage of each subcluster in control and pH group. **(C)** Dot plot representing ion transport genes and immune related genes in each subcluster of pillar cells. **(D)** The t-SNE plot showing the renames of subclusters in the pillar cells. The purple dotted circle indicated the ion cells, and the black dotted circle indicated the immune cells. **(E)** Violin diagram showing expression level of some immune related genes in the immune cells of pillar cells in the control and pH group.

In view of the important role of pillar cell in gills, its heterogeneity was analyzed to further understand the molecular mechanism under pH stress. Seven subclusters of pillar cells were identified (Figure 4A). The comparison of percentage of cells between control and pH groups showed that subcluster 0 and 3 were significantly increased, while subcluster 2, 4, and 6 were significantly decreased after pH stress (Figure 4B). Dot plot showed that ion transport genes such as CAs, VHAs, NCC and Rh were mainly expressed in subcluster 0 and 3, and immune marker genes such as crustins, ALF, C-type lectin, and proPO displayed high expressions in subcluster 2, 4, and 6 (Figure 4C). Based on this result, we redefined subcluster 0 and 3 to be ion cell subcluster, and subcluster 2, 4, 6 to be immune cell subcluster in pillar cells (Figure 4D). To further understand the functions of the immune cell subcluster in the pillar cells in response to high pH stress, some immune related genes were selected for violin plot display, and the results showed that the pH group exhibited high expressions of crustins, ALF, C-type lectin, proPO and Prx compared with the control group (Figure 4E). In addition, according to GO and KEGG enrichment analyses, DEGs in ion cell subcluster were mainly related to ion transport, and DEGs in immune cell subcluster were mainly related to Ribosome (Supplementary Figures S3, S4).

### Differentiation of pillar cells after pH stress

**FIGURE 5:**
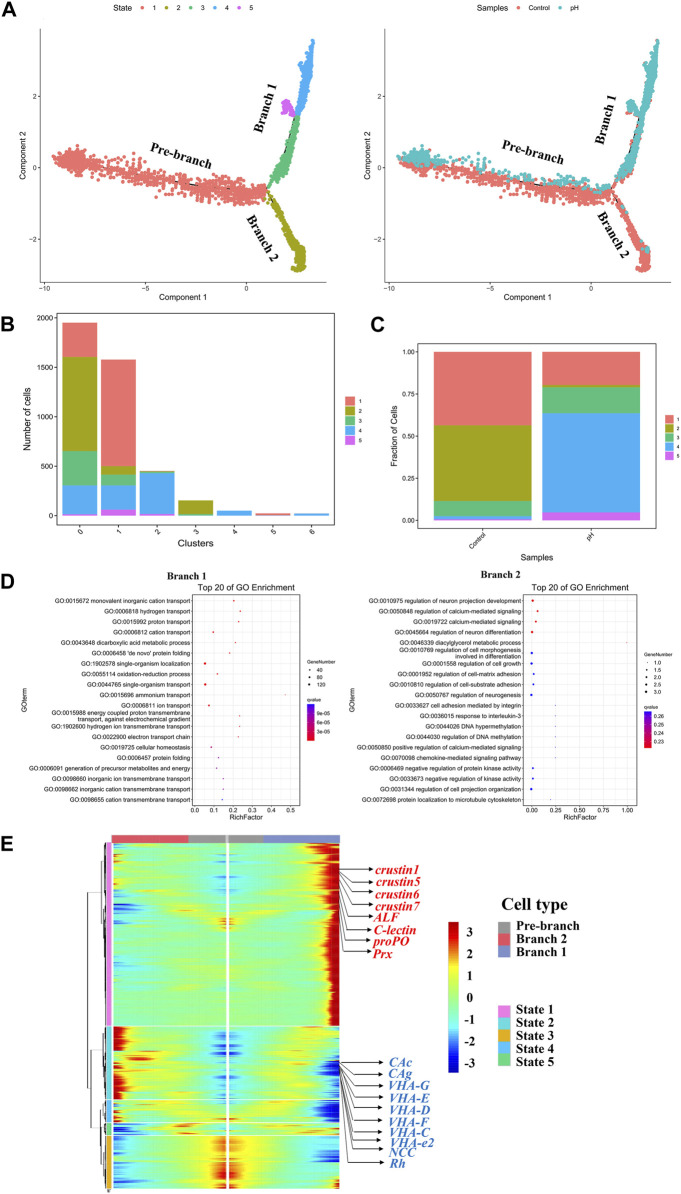
Reconstruction of the pillar cell subpopulation differentiation trajectory in a pseudotime manner. **(A)** Monocle 2 was used to track pillar cells in pseudotime using the scRNA-seq datasets. **(B)** The 7 subclusters of pillar cells distributed over 5 states of pseudotime differentiation trajectories. **(C)** Fraction of cells in the control and pH group distributed 5 states of pseudotime differentiation trajectories. **(D)** The GO enrichment of Branch 1 and Branch 2. **(E)** The pseudotime branch heatmap of pillar cells.

According to the change in trajectory, the pillar cells underwent three stages: the starting point of the branch (Pre-branch), Branch 1 (state 1–3,4,5) and Branch 2 (state 1–2). Notedly, the Branch 1 consisted mostly of the cells in pH group, while the Branch 2 consisted of the cells in control group (Figure 5A). State 2 was mainly located in the subcluster 0, state 1 was mainly located in subcluster 1, and state 4 was mainly located in subcluster 2 (Figure 5B). The state 1 and state 2 were mainly detected in the control group, while state 4 was mainly detected in the pH group (Figure 5C). This result was consistent with the new immune cell subcluster found in pH group. GO enrichment analysis revealed that the Branch 1 was significantly related to ion transport, including “monovalent inorganic cation transport”, “hydrogen transport”, “proton transport”, and “cation transport”. Regulation of cell differentiation and development related GO terms, such as “regulation of neuron projection development”, “regulation of calcium-mediated signaling”, “regulation of neuron differentiation”, and “regulation of cell morphogenesis involved in differentiation” were enriched in the Branch 2 (Figure 5D). A heatmap showing gene expression dynamics over pseudotime showed high expression of immune related genes and low expression of ion transport related genes in Branch 1 (Figure 5E).

### Weighted gene co-expression network analysis analysis of pillar cells

**FIGURE 6:**
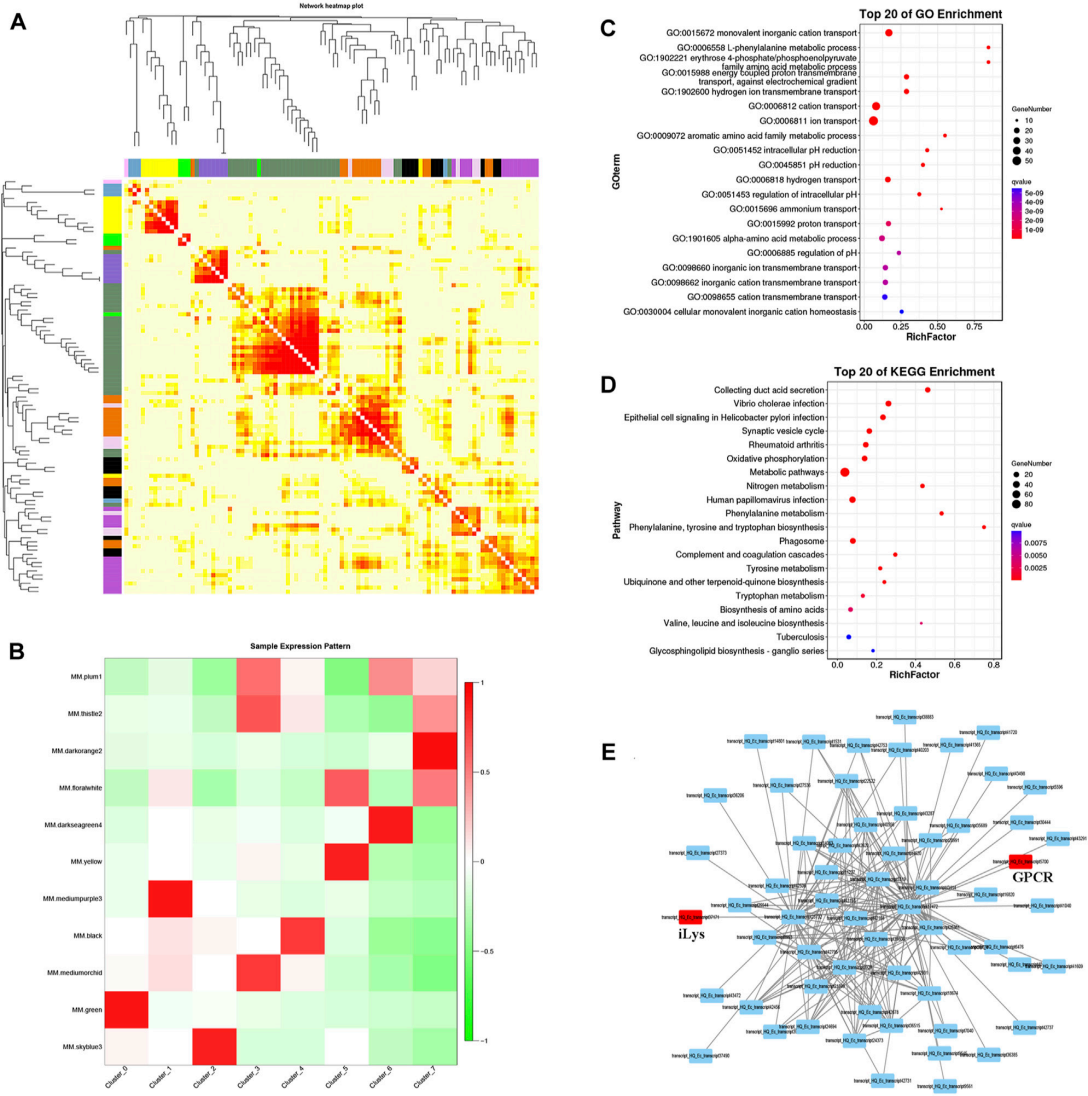
WGCNA analysis of pillar cell population. **(A)** Cluster the genes in the module and arrange the heat map. Each row and column represent a gene, and the darker the color of each point represents the greater the connectivity between the two genes corresponding to the row and column. **(B)** The heat map of sample expression pattern. Red represents high expression and green represents low expression. **(C)** GO enrichment of green module. **(D)** KEGG enrichment of green module. **(E)** Single-cell transcriptional networks inference of green module based on top 200 relationship gene pairs.

The correlation between the expression of each gene and the module eigenvalues was analyzed, and cluster the genes in the module and arrange the heat map, which indicated that the data from all cell clustered together naturally (Figure 6A). When scRNA-seq data were subjected to WGCNA, 11 gene-network modules were obtained (Figure 6B). Among the multiple modules, the green module is closest to the expression characteristics of the pillar cell population in terms of quantity and expression level. With the same strategy, we can easily assign different modules to different cells. For instance, the hemocyte population is mainly expressed by mediumpurple module and the septal cell can be represented by skyblue module. Here, we mainly focus on the pillar cell module to extrapolate the hub-gene networks of pillar cell population. When the pillar cell module was examined in combination with GO and KEGG analysis, major GO terms were related to ion transport including monovalent inorganic cation transport and hydrogen ion transmembrane transport (Figure 6C), and KEGG enrichment analysis revealed that pillar cell module was grouped into some ion transport-related pathways including collecting duct acid secretion, oxidative phosphorylation and nitrogen metabolism (Figure 6D). Meanwhile, we also identified the hub-gene network on pillar cell module and found two transcription factors, secretin family G-protein coupled receptor (GPCR) and invertebrate type lysozyme (iLys) (Figure 6E, red circle).

## Discussion

In aquatic animals, a rise in blood pH destroys the acid-base balance in the body and leads to respiratory alkalosis (Wilkie and Wood, 1996). In this study, hemolymph pH of *E. carinicauda* was also increased, which indicated that high pH stress caused shrimp acid-base disturbance. The effect of high pH stress on the gill cell structure was observed from the dramatic change in ultra-structures of the pillar cells and hemolymph space. These alterations may impair osmoregulation, acid–base regulation, ammonia excretion and respiration, and potentially lethal in shrimp with low tolerance to a changing environment (Mohamad et al., 2021). However, it is difficult to obtain an integrated scenario of the cellular and molecular mechanisms in response to stress by histology or bulk RNA sequencing methods. To address this issue, scRNA-seq was successfully performed in shrimp gills for the first time, which provided information on the cell clusters and their stress-activated states after pH stress. Importantly, we could clearly distinguish three important cell populations including pillar cells, hemocytes and septal cells, which are less well understood and for which marker genes were not previously available.

The pH stress affects ion regulation in aquatic crustaceans, whose primary site is the gill, with specific ion transporters expressed on some specific cells (Boudour-Boucheker et al., 2013; Ali et al., 2015). The pillar cells appear to be involved in ion transport, given their intimate connection with both the external medium and the hemolymph (Faleiros et al., 2010). Intralamellar septal cell, which only observed in phyllobranchiate gills, attached to both sides of the pillar cells, and function together to assure ion and gas exchange across the gill epithelium (Thabet et al., 2017). In this study, the GO enrichment analysis of pillar cells and septal cells were mainly related to ion transport, which fully confirmed the ion transport function of these two cell types. Base on the previous research, VHA immunofluorescence was localized in the membranes of the apical evaginations and in clustered subapical areas of pillar cells, and NKA labeling was restricted to the septal cells (Boudour-Boucheker et al., 2014). In this study, most ion-transport genes including VHAs, CAs, Rh, NCC, NHE3, NKCC, and NBC identified in the pillar cells of gills in *E. carinicauda*, therefore, except VHAs, other genes such as CAs, Rh, NCC, NHE3, NKCC and NBC, could also be considered as molecular marker candidates for pillar cells in shrimp gills. In addition, NKA was identified in septal cells of *E. carinicauda*, which was similar with the location of NKA in *Macrobrachium amazonicum*. These results suggest that morphologically different cell types within the gill lamellae may also be functionally specialized. It was proposed that pillar cells absorbed ions (Cl^−^, Na^+^, HCO_3_^−^, etc.) by specific ion-transport genes (VHAs, NCC, CAs, Rh, etc.) that were transported either directly to the hemolymph space or through a junctional complex to the septal cells, which may be responsible for active Na^+^ delivery to the hemolymph through NKA (Boudour-Boucheker et al., 2013). This suggests an ion transport functional link between septal cells and pillar cells in osmoregulation and acid-base regulation in gills of *E. carinicauda*. In addition, shrimp, as invertebrates, have an open vasculature that allows circulating hemocytes to infiltrate the tissues, where they are referred to as sessile hemocytes (Koiwai et al., 2018). Coincidentally, we also identified the sessile hemocyte cluster in the gill tissue of *E. carinicauda* by scRNA-seq, which expressed many immune-related genes.

In this study, the pillar cells had the most numerous of DEGs between control and pH group, with down-regulated expression of ion transport related genes and up-regulated expression of immune related genes. The pillar cells changed significantly at the cellular and molecular level after high pH stress, which could be regarded as the target cell type of shrimp gills in response to high pH stress. In fish, the high pH stress leaded to the decrease of H^+^ concentration in fish, thus the quite toxic ammonia nitrogen (NH_3_) cannot be combined with H^+^ and cannot be discharged in the form of relatively non-toxic ammonium (NH_4_^+^) *in vivo* (Zhao et al., 2020). In addition, higher pH promoted a higher proportion of CO_3_^2-^ resulting in higher toxicity (Yao et al., 2016). Therefore, it can be speculated that high pH stress could also significantly affect the ion transport such as H^+^, NH_4_^+^, CO_3_^2-^ in shrimp. VHAs play a critical role in maintaining cellular pH homeostasis through a proton pumping activity and are important proteins in the “Oxidative phosphorylation” and “Collecting duct acid secretion” pathway (Venediktova et al., 2013; Xing et al., 2019). Carbonic anhydrases play important role in keeping balance of body acid-base and regulating the pH of body fluid by catalyzing the interconversion of CO_2_ and HCO_3_^−^, and are important members in the “Nitrogen metabolism” pathway (Liu et al., 2015). Rh glycoproteins are a family of membrane proteins, which facilitate NH_3_ diffusion across the gills (Nakada et al., 2010). In addition, the process of NH_3_/NH_4_^+^ transport, H^+^ excreting or HCO_3_^−^/CO_3_^2-^ transport are often accompanied by Na^+^/Cl^−^/K^+^ ion transport, which are regulated by NCC, NHE3, NKCC or NBC (McNamara and Faria, 2012). Therefore, the down-regulation of ion (H^+^, NH_4_^+^, CO_3_^2-^, etc.) transport genes in pillar cells suggested that the suppression of ammonia excretion and H^+^ transport resulted in acid-base disturbance of during ambient high pH exposure in *E. carinicauda*. The reason leading to a decrease in the transcription of related ion transport proteins may be microstructure changes of gill morphology after high pH stress.

In view of the distinct patterns of ion transport and immune gene expressions in pillar cells after high pH stress, we speculated cell heterogeneity of pillar cells induced by pH stress. Further, we identified six subclusters of pillar cells and found a new immune cell subcluster in the pillar cells after high pH stress. Notedly, many immune related genes such as crustins, ALF, proPO were up-regulated in the pH group, which indicated that some pillar cells were in the immune-activated states after high pH stress. Although previous studies reported that the immune defense function of gills in crustacean was considered to be related to the plentiful hemolymph in this tissue, and we did identify the sessile hemocyte cluster of the gills in this study, there is no information about immune response function of the pillar cells (Wang et al., 2018; Lv et al., 2020). This is the first report on the immune function of pillar cell except for ion transport. In addition, GO and KEGG enrichment analysis of DEGs in the immune cell subcluster revealed that “Ribosome” was significantly enriched. The ribosome is a complex ribonucleoprotein-based molecular apparatus that is responsible for the synthesis of proteins with cells (Jin et al., 2021). Recent work has shown that the synthesis of many defence proteins is required in stress response, and that ribosomal translation is crucial for maintaining timely synthesis of stress defence proteins (Zhu and Dai, 2020). For example, a large proportion (21.8% in blood and 33.3% in hepatopancreas) of ribosomal genes was significantly upregulated in cells of *P. monodon* after ammonia-N stress (Li et al., 2022). The ribosome translation should be studied in parallel with traditional transcriptional regulation in the future to better understand shrimp stress defences. Therefore, we speculated that immune cell subcluster is the main effective cell population of immune response and stress defence in pillar cells after high pH stress. In addition, the pseudotime analysis of pillar cells was performed, and the Branch 1 consists of most cells in the pH group. Notedly, the DEGs involved in ion transport were significantly down-regulated, and DEGs involved in immune response were significantly up-regulated in Branch 1, which was consistent with the results of comparative single-cell transcriptome analysis between control and pH group. This funding showed that some of the pillar cells converted to ion cell types with suppressed ion transport function, and some of the pillar cells converted to immune cell types with active immune response function after high pH stress. These results demonstrated the functional heterogeneity of pillar cells in shrimp gills after high pH stress.

The ion transport system is characterized by gene regulatory network. However, the ion transport network and mechanism of aquatic animals remains largely unknown. The WGCNA was used to infer gene associations within the pillar cell population to further understand its ion transport regulatory network. We divided all the single cells into different modules and chose green as the representative of pillar cell based on the distribution of pillar cell-related clusters. As expected, the GO and KEGG enrichments showed that it mainly participated in ion transport, which was consistent with pillar cell effector functions. In the network interaction map, two transcription factors were screened, which might be important to regulate the activity of pillar cells. GPCRs are ancient, ubiquitous, constitute the largest family of transducing cell surface proteins, and are integral to cell communication *via* an array of ligands/neuropeptides (Tran et al., 2019). The secretin family (class B) GPCRs are involved in stress response, including hormones, neurotransmitters, glucocorticoids, steroids, lipids, nucleotides, peptides, and ions (Techa et al., 2023). Lysozymes are widely distributed immune effectors exerting muramidase activity against the peptidoglycan of the bacterial cell wall to trigger cell lysis and iLys are only found uniquely in invertebrates (Zhou et al., 2017). Several immune-related transcription factor binding sites were discovered in the putative promoter region of iLys (Bathige et al., 2013). The exact roles of secretin family GPCR and iLys in the regulation of pillar cell activity will be clarified in further research.

In summary, on account of hemolymph acid-base disturbance and dramatic change of gill cells caused by high pH stress, scRNA-seq were performed to deeply study the shrimp gill cell response mechanism after high pH stress. The data revealed that seven cell clusters were successfully classified, and three main cell types were identified based on specifically expressed marker genes. Some important ion transport genes such as CAs, Rh, NCC, and VHAs can be used as gene markers to identify pillar cells. After pH stress, the pillar cells are the main target cell population of ion regulation characterized by suppression of hydrogen transport and nitrogen compound transport. We further identified a new immune subcluster in pillar cells with up-regulated expressions of the immune responsive and ribosome genes, which illustrated the functional heterogeneity of pillar cells in response to high pH stress. The pseudotime analysis showed that the pillar cells could differentiate into ion cell types and immune cell types after high pH stress. The regulatory network of pillar cell population was predicted by WGCNA and two transcription factors were identified. Altogether, this scRNA-seq analysis documents the diversity of shrimp gill cell populations and provides a comprehensive resource of gene expression profiles for understanding the functions of gill cells after high pH stress.

## Data Availability

The data presented in the study are deposited in the NCBI repository, accession number PRJNA882579.

## Supplementary material

SUPPLEMENTARY FIGURE S1 GO enrichment of the DEGs in Cluster 0, 1 and 2.

SUPPLEMENTARY FIGURE S2 The relative expression of ion transport related genes in gills.

SUPPLEMENTARY FIGURE S3 GO and KEGG enrichments of DEGs in ion cell subcluster of pillar cells.

SUPPLEMENTARY FIGURE S4 GO and KEGG enrichments of DEGs in immune cell subcluster of pillar cells.
