# Supplementary figures and images for: Highly sensitive and specific responses of shrimp gill cells to high pH stress based on single cell RNA-seq analysis
Source: Front Cell Dev Biol. 2022 Nov 8;10:1031828. doi: 10.3389/fcell.2022.1031828 (PMC9679296; doi:10.3389/fcell.2022.1031828)

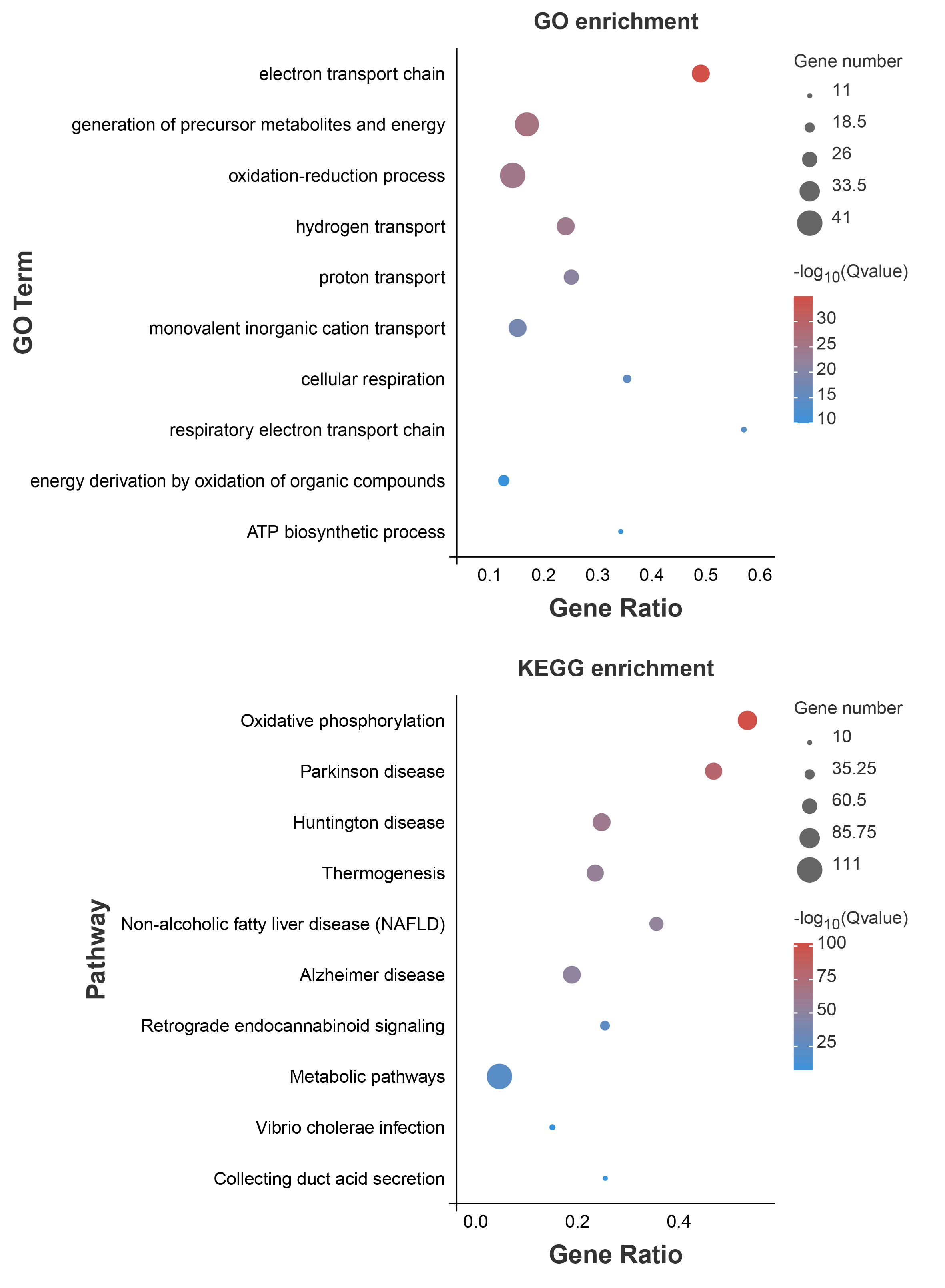

Supplement: Supplementary file 1 [file Image3.TIF]

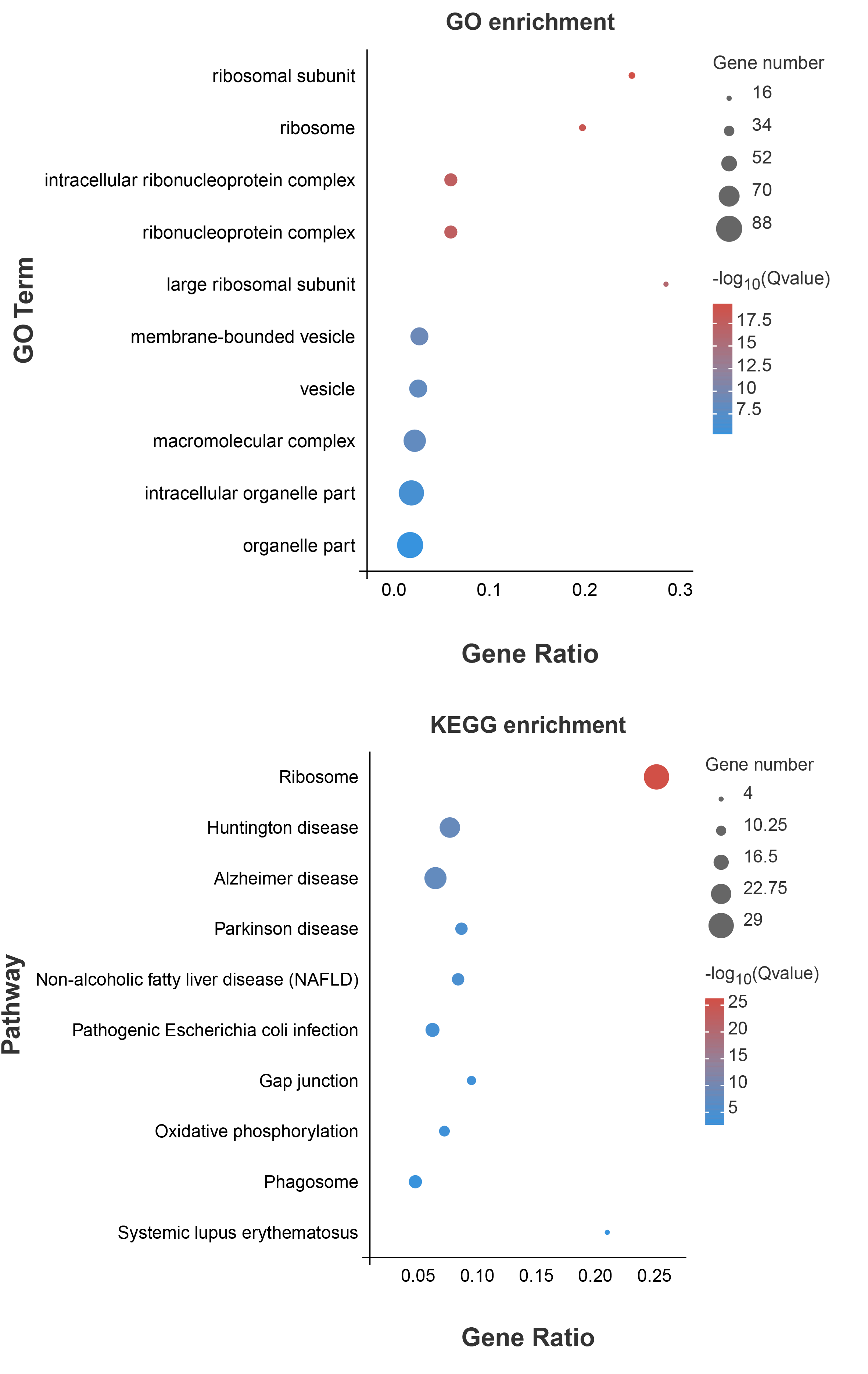

Supplement: Supplementary file 2 [file Image4.TIF]

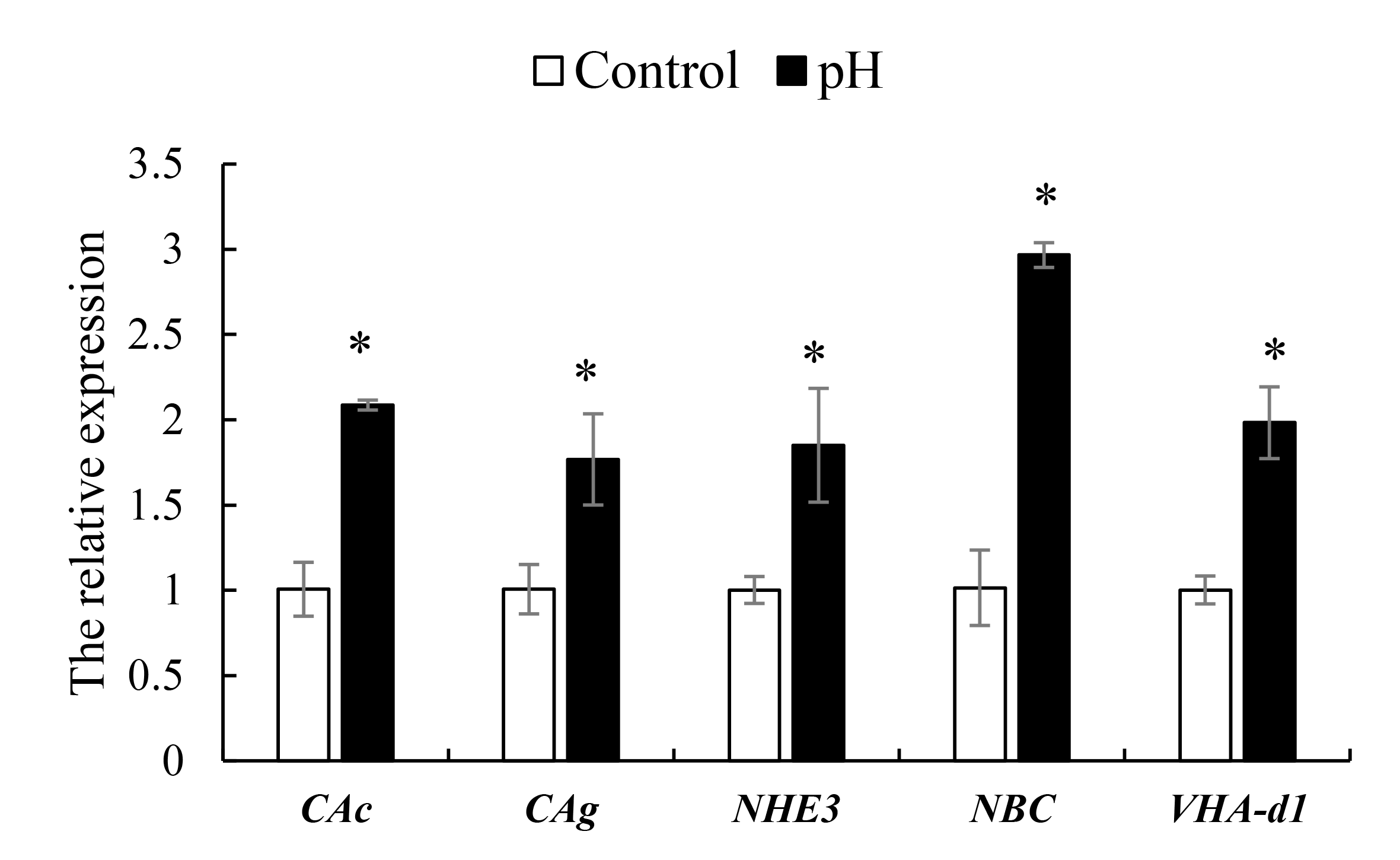

Supplement: Supplementary file 3 [file Image2.TIF]

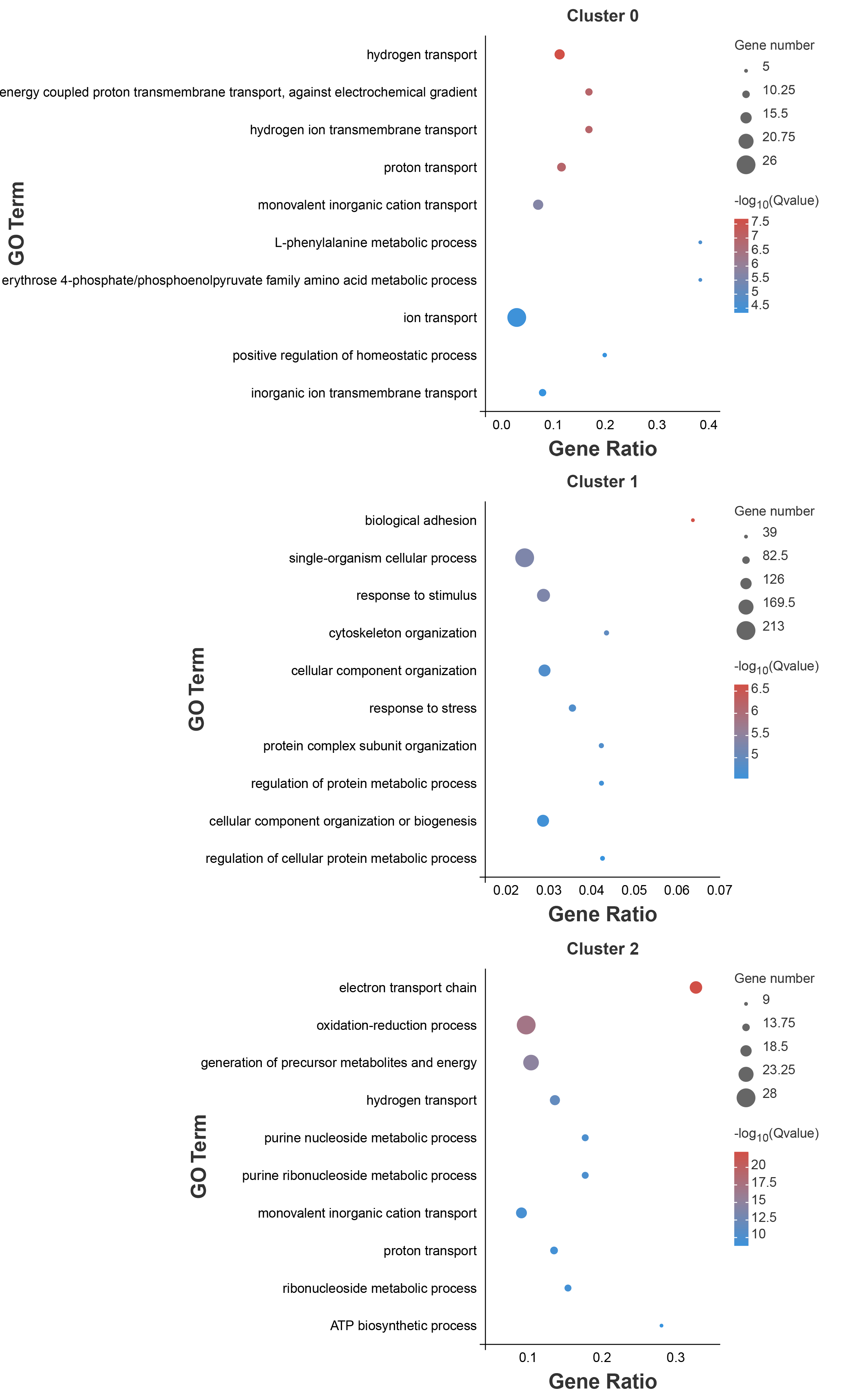

Supplement: Supplementary file 4 [file Image1.TIF]
